# Vesicovaginal reflux: a case report and literature overview

**DOI:** 10.3389/fped.2026.1849196

**Published:** 2026-05-11

**Authors:** Yuming Wang, Shuai Sun, Lei Geng, Yewen Wang, Gaofeng Li, Xiaoliang Xu

**Affiliations:** 1 Department of Pediatric Surgery, Binzhou Medical University Hospital, Binzhou, China; 2 Department of Ultrasound, Binzhou Medical University Hospital, Binzhou, China

**Keywords:** hydrocolpos, ultrasound, urethrovaginal reflux, urinary incontinence, vesicovaginal reflux

## Abstract

Vesicovaginal reflux (VVR) is a functional voiding disorder characterized by the retrograde flow of urine into the vagina during micturition, often presenting with daytime incontinence or postvoid dribbling. It is commonly underrecognized and may be mistaken for anatomical abnormalities such as vesicovaginal fistula or obstructive hydrocolpos, which require surgical intervention. We report a case of an 8-year-old girl who presented with dysuria, urinary urgency, and fever. Transabdominal and transrectal ultrasounds revealed an anechoic area measuring 7.3 × 3.3 × 4.0 cm in the vagina while urinary bladder was filled, which disappeared after voiding. Pelvic magnetic resonance imaging showed a cystic fluid signal between the bladder and rectum that resolved following urination. Voiding cystourethrography ruled out filling defect, which suggested bladder-vaginal reflux. A literature search of PubMed and Google Scholar from 1972 to the present using the terms “urinary incontinence,” “hydrocolpos,” and “vesicovaginal reflux” identified 13 relevant articles. Including the present case, a total of 58 cases of VVR were reviewed, ranging from children to middle-aged adults. The diagnosis of VVR should be considered when transient fluid collection is observed in the vagina on ultrasound and disappears after complete voiding. Treatment focuses on behavioral therapy, including proper voiding posture and toileting habits, along with weight control when indicated.

## Introduction

Urinary incontinence is a major health issue affecting individuals of all ages and a common reason for seeking medical care, with particular prominence in the pediatric population. Daytime urinary incontinence is not an uncommon phenomenon in children. The prevalence of daytime wetting is known to be higher in girls aged 6 to 12 years, approximately ranging from 3.1% to 9.5% (1, 2). The most common underlying causes of urinary incontinence in children include bladder-bowel dysfunction, overactive bladder, delayed urination, and dysfunctional voiding (3). Among its causes, Vesicovaginal reflux (VVR) is a relatively uncommon one (4). VVR is a functional voiding disorder characterized by the reflux of urine into and its retention within the vagina through the vaginal orifice, in the absence of clear anatomical abnormalities or neurogenic pathologies. This condition is more frequently observed in adolescent females with urinary incontinence (5), but it can also occur in middle-aged women (6). In clinical diagnosis, it is essential to first rule out anatomical abnormalities, such as an ectopic ureteral opening into the vagina, labial adhesions, or vesicovaginal fistula (the latter being more common in elderly women with a history of pelvic surgery or radiotherapy) (7). Only after excluding these organic pathologies can a diagnosis of functional VVR be considered. Treatment primarily focuses on behavioral modification, health education, and urination hygiene training (5). This paper reports a case of a child in whom an anechoic fluid collection (vaginourinary pouch) was detected within the vagina during transabdominal ultrasound examination.

## Case report

An 8-year-old girl was admitted due to “dysuria for 3 days,” accompanied by urinary urgency and fever (38.5°C). Ultrasound performed at another hospital suggested cystitis and possible urethral diverticulum. Her symptoms improved after anti-infective treatment. She presented to our hospital for further evaluation of the suspected urethral diverticulum. Physical examination revealed a body temperature of 36.6°C, height of 142 cm, and weight of 50 kg, BMI was 24.79. The abdomen showed no tenderness or rebound tenderness, and no percussion pain was noted in the bilateral renal areas. The urethral and vaginal openings were normal and there was no vaginal discharge. Renal function, white blood cell count, and urinalysis were unremarkable.

**Figure 1:**
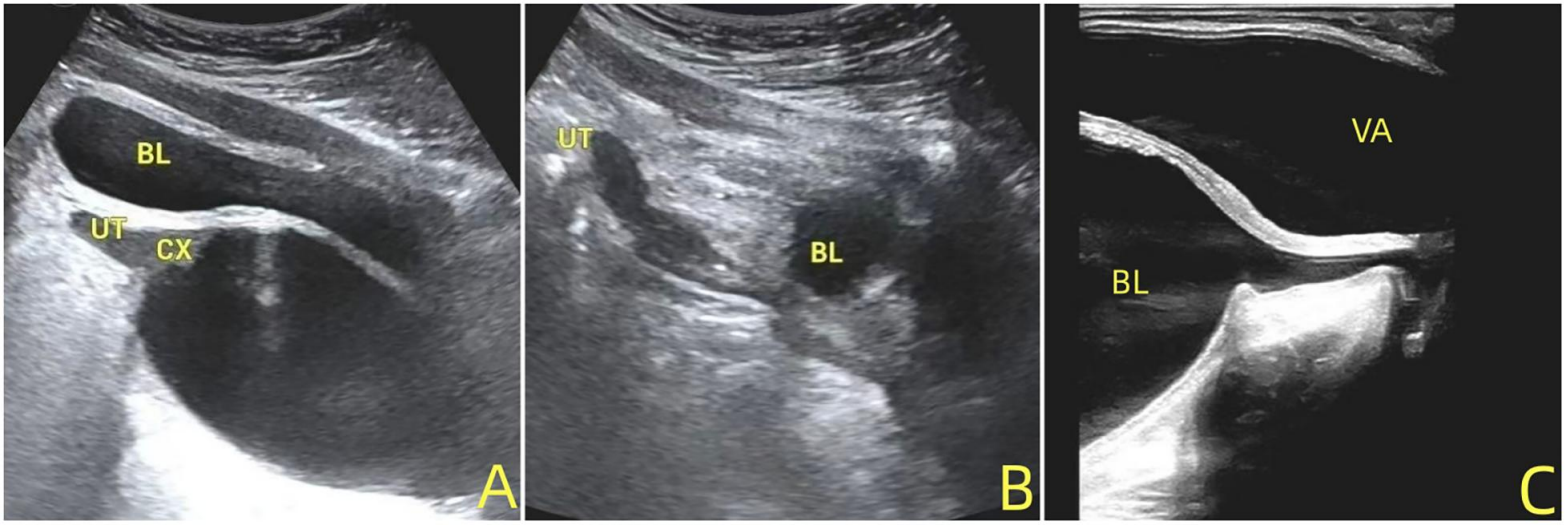
**(A)**: transabdominal ultrasound shows a large anechoic fluid collection distending the vagina before voiding; **(B)**: transabdominal ultrasound shows disappearance of the anechoic fluid collection in the vagina after voiding; **(C)**: transrectal ultrasound shows a large anechoic fluid collection distending the vagina before voiding. BL, Bladder; UT, Uterus; VA, Vagina.

Transabdominal and transrectal ultrasound showed (Figure 1) that an anechoic area measuring approximately 7.3 cm × 3.3 cm × 4.0 cm was detected in the vagina while sufficient bladder was filled. The bladder wall and vaginal wall exhibited good continuity, with no clear communication observed. During dynamic observation, occasional separation of the urethra was noted, with a urethral length of approximately 2.8 cm and no significant continuity interruption. After instructing the child to simulate urination, the urethra dilated, revealing communication between the urethral and vaginal openings, and the anechoic area in the vagina subsequently disappeared. Both kidneys and ureters ultrasonography were unremarkable. The initial diagnosis of urinary bladder-vaginal reflux was considered.

**Figure 2:**
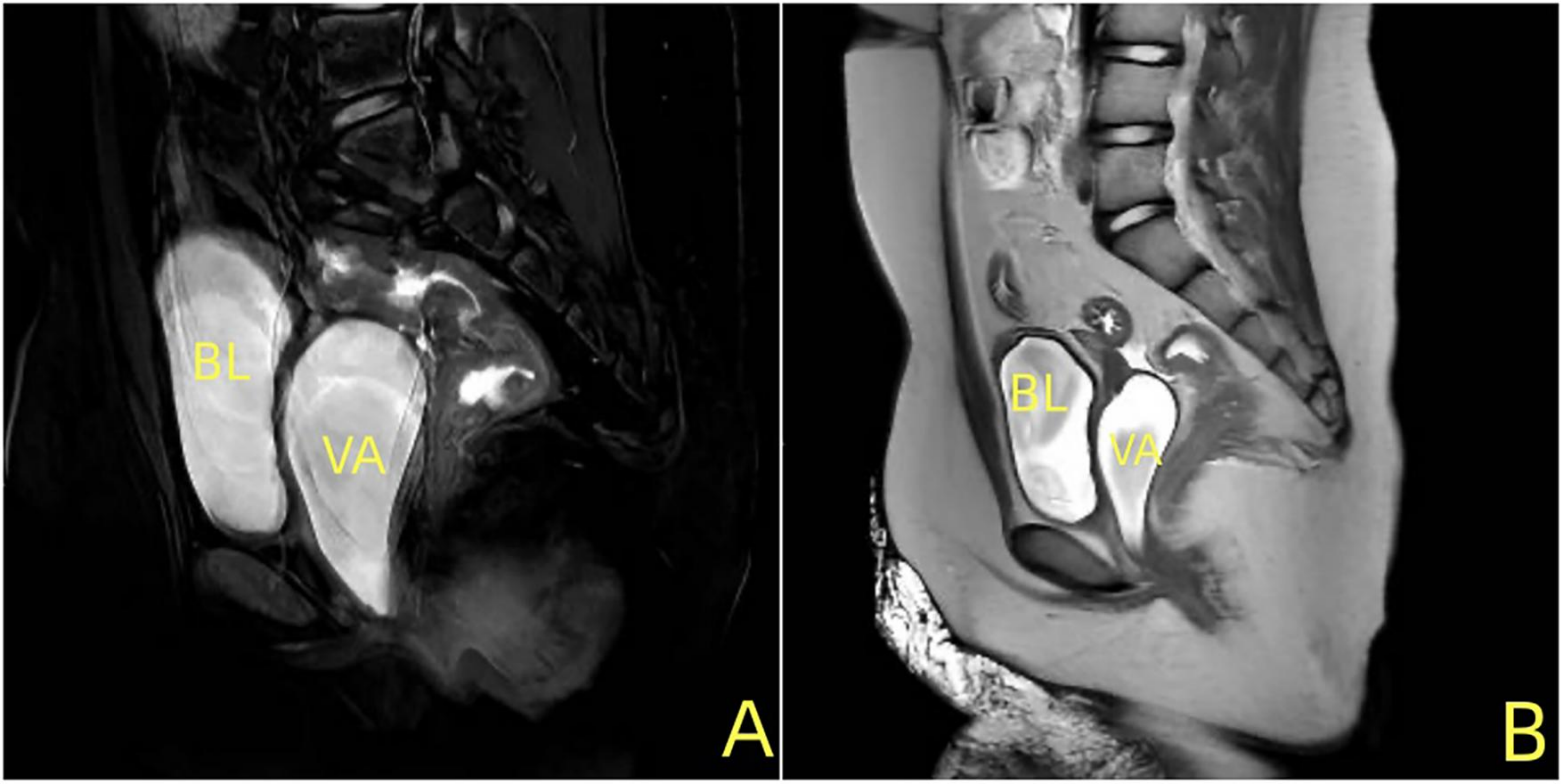
Sagittal magnetic resonance imaging (MRI) shows the presence of a fluid signal with homogeneous density in the bladder and vagina.

**Figure 3:**
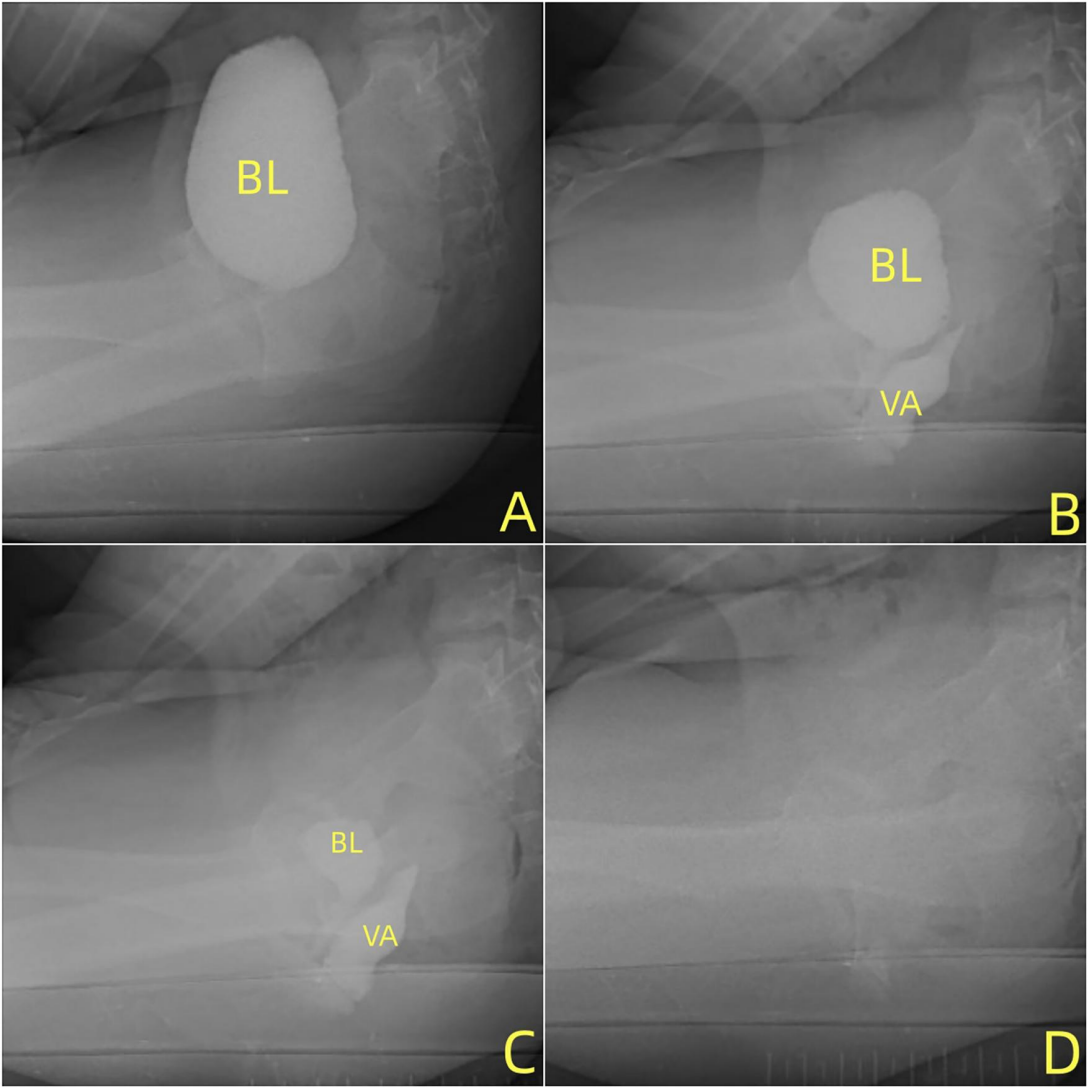
(**A→D**): voiding cystourethrography shows that the fluid within the bladder decreases after voiding, while the fluid within the vagina increases. Both the bladder and vaginal fluids disappeared after completion of voiding.

Pelvic magnetic resonance imaging (MRI) was indicated (Figure 2). Under the state of urine filling, a cystic band-like fluid signal was observed between the bladder and rectum.Voiding cystourethrography (VCUG) showed no filling defects. During spontaneous urination, a strip-like contrast filling, approximately 7 cm in length, was observed behind the bladder-urethra (Figure 3). The vaginal fluid collection completely disappeared during urination, suggesting bladder-vaginal reflux.

The child currently has no urinary system symptoms through proper voiding guidance, and behavioral modification. Weight control was advised. After six months of follow-up, the child's BMI was 22.30, and she had no further dysuria or other discomfort.

## Discussion

Although VVR is a cause of urinary incontinence in school-aged girls, it is often underdiagnosed, and current literature reports are very limited. A computerized search of PubMed and Google Scholar was performed for relevant literature from 1972 to the present using the search terms “urinary incontinence,” “hydrocolpos,” “urethrovaginal reflux”and “vesicovaginal reflux.” We reviewed 13 articles along with the case reported herein, identifying a total of 58 cases of vesicovaginal reflux across all age groups from children to middle-aged adults (4–16).

The reported incidence of VVR varies across studies, primarily due to its low diagnostic rate and high underreporting. Among prepubescent girls presenting with symptoms of urinary leakage, approximately 12% to 15% of urinary incontinence cases are attributed to vaginal reflux (9, 17).

The diagnostic criteria for VVR proposed by Bernasconi et al. (18) are as follows:

1. Moderate urinary incontinence occurs within 10 minutes after normal urination;
2. Absence of other voiding symptoms (including urinary frequency or infrequent urination, dysuria, weak, interrupted, or discontinuous urinary stream, and the sensation of incomplete bladder emptying despite normal post-void residual urine);
3. Uroflowmetry shows a smooth bell-shaped curve without interruption or intermittent patterns;
4. Electromyography of the external urethral sphincter demonstrates minimal or absent electromyographic activity during urination.

In affected girls, the anatomical appearance of the urethral meatus and external genitalia corresponds to their age (9). The exact pathogenesis of VVR has not been fully elucidated, and several possible mechanisms have been proposed.

1. Developmental factors related to bladder position: The relatively high position of the bladder during the neonatal and infant periods may predispose to VVR. With growth and physical development, the bladder descends, and changes in urethral length and position lead to a progressive decrease in the vulvar-urethral angle (VUA), thereby reducing the incidence of VVR (6). Consequently, the prevalence of VVR gradually declines during prepubertal development and is expected to become rare in adulthood (5).
2. Local anatomical factors: In young girls, the urethral meatus is in close proximity to the vagina, and the hymenal ring, labia minora, and labia majora are small and tightly positioned. Even in the absence of anatomical adhesions, the labial folds can sometimes form a valve-like structure that directs the urinary stream posteriorly into the vagina. A similar situation can occur in obese patients, where abundant adipose tissue in the groin and thighs causes the labia to adhere closely, leading to urine reflux into the vagina (10). Among the previously reported overweight patients (Table 1) as well as our patient, weight reduction therapy was recommended.
3. Pelvic floor muscle dysfunction: Overactivity or dysfunction of the pelvic floor muscles (pelvic floor muscle spasm) may also contribute to the occurrence of VVR (18).

The diagnosis of VVR is often challenging because its symptoms are frequently mixed with or masked by other lower urinary tract storage or voiding symptoms. The leakage experienced by affected children is more commonly attributed to overactive bladder or voiding dysfunction, leading to inappropriate treatment. In some children, leakage most frequently occurs when they begin to walk. Others may experience their first episode of urine extrusion during laughter or coughing due to increased abdominal pressure, a manifestation easily mistaken for stress urinary incontinence (9). In cases of VVR, the characteristic history is that the child reports their underwear becoming wet within 5 to 10 minutes after urination, particularly when standing up from a seated position and starting to walk. Missing this key aspect of the history can easily lead to a deviation in diagnostic focus and result in misdiagnosis (17).

**Table 1:** Review Of literature on VVR in children and adults,including age, symptomatolgy, imaging findings, and management.

**Reference**	**Age (years)**	**Symptomatolgy**	**Imaging Findings**	**Management**
Muneer Fazea et al. (4)	37	Abdominal pain, Daytime incontinence	US:Anechoic mass posterior to bladder, anterior displacement and posterior acoustic enhancement. CT:Distended fluid-filled vagina on full bladder imaging, suggesting urocolpos.	Levofloxacin 500 mg daily for 5 days; behavioral therapy with voiding retraining program (including proper toileting position).
Sumitra Reddy et al. (5)	18	Excessive menstruation	US:large fluid-filled cystic lesion in midline pelvis, distending vagina; uterine corpus and cavity normal.	Behavioral therapy and toilet training.
Sumedha Arora et al. (6)	22,30	Case 1:Irregular menses. Case 2:Occasional urinary dribbling.	Case 1:US: large anechoic collection (12.2 × 9.1 × 7.9 cm) distending the vagina. Case 2:US: cystic lesion (7.4 × 1.8 × 4.1 cm) in vagina posterior to distended urinary bladder.	Case 1: NA. Case 2:Weight reduction.
Damien Motavasseli et al. (7)	47 ± 15 years (16 cases)	Postmicturition incontinence.	Cystoscopy and CT urography in all 16 patients: no abnormalities (fistula, diverticula, urethrocele, ectopic ureter).	Specific maneuvers (intravaginal finger insertion in cross-legged position) or intravaginal tampon; pelvic floor exercises.
Gamze Kilicoglu et al. (8)	5, 11, 13	Case 1:Daytime incontinence, postvoid dribble. Case 2:Daytime wetting. Case 3:Frequency, urgency, daytime wetting.	US: Fluid-filled mass posterior to bladder, disappearing after voiding (all three). MRI (Case 2): Confirmed urine filling of vagina after voiding.	Case 1 and 2:Behavioral therapy with voiding retraining. Case 3 (labial adhesions): Surgical adhesiolysis; topical estrogen cream ×2 weeks; antibiotics for UTI.
Sven Mattsson et al. (9)	7–15 (21 cases)	Daytime leakage, wetting 5–10 min after voiding.	NA	Behavioral therapy with voiding retraining.
María Fernández-Ibieta et al. (10)	9,10,10	Case 1 and 3:Diurnal incontinence. Case 2:Incomplete voiding.	Case 1:VCUG:Progressive gross vaginal distension (urocolpos) due to retrograde filling as bladder empties. Case 2:VCUG: VVR. Case 3:VCUG:VVR with small urocolpos.	Behavioral therapy (all three).
Monali Warade et al. (11)	14	Dribbling.	US:Grossly distended fluid-filled vagina, suggestive of hydrocolpos.	NA
Ankit Balani et al. (12)	15	Intermittent dysuria, vulvovaginitis.	US: Anechoic cystic lesion posterior to distended urinary bladder, outlining the cervical os, with mild mass effect (superior uterine displacement and anterior bladder displacement).	Behavioral therapy with voiding retraining.
Venkatraman Indiran et al. (13)	16	Pelvic and loin pain.	CT and MRI:Bilateral pelviureteric junction obstruction and urocolpos; no defect/communication between bladder and vagina.	Behavioral therapy with voiding retraining.
Sachin Somwanshi et al. (14)	13	Recurrent UTI, vague urge incontinence.	US and CT:Grossly distended fluid-filled vagina, suggestive of hydrocolpos.	Proper toilet training.
Emma M. Snyder et al. (15)	5, 6, 10, 12	UTI,precocious puberty,nonspecific pelvic pain.	US:Variably distended fluid-filled vagina outlining cervical os.	NA
By Christine Butcher et al. (16)	5,6	Case 1:Post micturition dribble. Case 2:Post micturition dribble, nocturnal enuresis.	VCUG (both): Vaginal filling during micturition, considerable contrast retention at end, draining slowly (some still present at 20 min).	NA
The present case	8	Dysuria，urinary urgency， fever.	US:anechoic area (7.3 × 3.3 × 4.0 cm) in vagina after bladder filling. MRI:Cystic band-like fluid signal between bladder and rectum, disappearing after urination. VCUG:Suggesting bladder-vaginal reflux.	Weight control,behavioral therapy with voiding retraining.

US, Ultrasound; CT, Computed tomography; MRI, Magnetic Resonance Imaging; UTI, Urinary Tract Infection; VCUG, Voiding Cystourethrography; NA:Not Available.

Therefore, the key to diagnosis lies in conducting a comprehensive and appropriate history-taking. Through detailed inquiry, children with such chronic conditions can be relatively easily distinguished from those with other functional voiding disorders (such as nocturnal enuresis, overactive bladder, voiding postponement, or giggle incontinence), and also differentiated from patients with serious genitourinary disorders (such as neurogenic bladder or ectopic ureter located below the distal sphincter) (19). Detailed history-taking should also include a frequency-volume chart for at least three consecutive days, recording urine output, fluid intake, and episodes of urinary leakage or damp underwear. This helps provide a good understanding of functional bladder capacity, abnormal drinking habits, the presence of polyuria, and the severity of voiding issues. A thorough pelvic and perineal examination is strongly recommended to rule out any abnormalities of the external genitalia, such as hypospadias, imperforate hymen, or labial adhesions (9, 11, 15).

Hematological and urine tests help rule out urinary tract infections. Urinary flow rate measurement and post-void residual urine volume assessment can identify potential concomitant bladder dysfunction.The preferred initial imaging modality is ultrasound, which demonstrates vaginal distension due to fluid during the full bladder phase. This finding is transient and resolves after voiding. MRI is a reasonable next step to exclude uterine anomalies, septa, bicornuate uterus, and associated Müllerian anomalies (1, 5, 6). CT has a high spatial resolution and can detect a small fistula between the vagina and urinary bladder or an ectopic ureteric insertion. It has the limitation of causing exposure to ionizing radiation and hence, is less preferable in young patients (6). In ultrasound imaging, fluid accumulation in the vagina (vaginal urine pooling) is a typical finding, though the disappearance of such fluid after some time may confuse inexperienced radiologists. If the child is not scheduled for a follow-up ultrasound, this key sign may be overlooked. When uncertainty exists, direct observation of the voiding process under real-time ultrasound monitoring (including leg crossing to induce urethrovaginal reflux and full leg separation to promote complete emptying) can confirm the diagnosis (8).

Voiding cystourethrography (VCUG) often reveals urethrovaginal reflux (20). When performed by a urologist using the correct technique, this examination is valuable for diagnosing VVR. VCUG can show gradual vaginal distension during voiding due to retrograde urine flow as the bladder empties. Typically, the voiding phase images in VCUG are not reviewed in detail, as the focus is often on excluding anatomical bladder abnormalities and vesicoureteral reflux. In all such cases, post-void images should be captured (11). Compared with VCUG, contrast-enhanced ultrasonography is not only more sensitive but can also detect higher grades of reflux. Moreover, it does not use ionizing radiation, which is especially important in pediatric patients (21).

This type of reflux in girls can lead to genital irritation, odor, and abnormal vaginal discharge, and may also predispose them to lower urinary tract infections. Approximately 40% of children with VVR have a history of urinary tract infections (22). Among the previously reported cases (Table 1), a total of 7 cases (including our patient) had definite symptoms of urinary tract infection.The condition is often associated with vulvar itching and recurrent vulvovaginitis, which can usually be effectively alleviated with simple measures. Vaginal reflux can also cause bacterial contamination of urine samples, making treatment more challenging and time-consuming (17, 18).

The focus of treatment is behavioral therapy, typically focuses on establishing proper voiding habits (6, 20). The foundation lies in excluding other anatomical and neurological causes and re-establishing a regular voiding pattern through adequate hydration, hygiene maintenance, and scheduled urination. As mentioned earlier, adopting the correct posture and fully spreading the legs during urination to prevent urine from flowing backward into the vagina is crucial. Human anatomy is inherently suited for elimination in a squatting position (23), but modern technology has changed our approach. Traditional toilets position the hips and knees at a 90-degree flexion, which is even more pronounced when the toilet is high, such as when used by children (17). This elevated posture hinders the complete relaxation of the muscles responsible for urination or defecation. When the thighs and legs are not sufficiently abducted and relaxed, VVR is further promoted (24). Placing a footrest in front of the toilet can help support the feet of young girls. Among the previously reported cases (Table 1), all remaining patients received behavioral therapy with voiding retraining, except for one case with labial adhesions that required surgical separation. Patients with vulvovaginitis and urinary tract infections caused by VVR should be treated with proper antibiotics (6).

## Conclusion

Vesicovaginal reflux (VVR) is an easily overlooked functional voiding disorder that can occur across all age groups from childhood to middle age. Its clinical manifestations often include post-void dribbling, daytime urinary incontinence, or recurrent urinary tract infections. The key to diagnosis lies in increasing awareness of the condition through comprehensive clinical history-taking, physical examination, and appropriate imaging evaluation. Ultrasound is the preferred imaging modality, and the characteristic finding of VVR is an anechoic collection in the vagina on a full bladder that disappears after voiding. Voiding cystourethrography and magnetic resonance imaging are useful for excluding anatomical abnormalities. Behavioral therapy is the cornerstone of treatment, including instruction on proper voiding posture (e.g., wide abduction of the legs during voiding and the use of a footstool when sitting on a toilet), establishing regular voiding habits, and weight control. Most patients achieve satisfactory relief with non-surgical measures. Increased awareness of this condition among clinicians and radiologists is essential to avoid misdiagnosis as a surgical emergency or unnecessary invasive procedures, thereby reducing patient anxiety and healthcare resource waste.

## Data Availability

The raw data supporting the conclusions of this article will be made available by the authors, without undue reservation.
